# Furosemide Reduces Radionuclide Activity in the Bladder in ^18^F-PSMA-1007-PET/CT: A Single-Center Retrospective Intra-Individual Comparative Study

**DOI:** 10.3390/diagnostics15151931

**Published:** 2025-07-31

**Authors:** Martin A. Cahenzli, Andreas S. Kreusch, Philipp Huber, Marco Dressler, Janusch P. Blautzik, Gregor Sommer

**Affiliations:** 1 Institute of Radiology and Nuclear Medicine, Hirslanden Klinik St. Anna, 6006 Lucerne, Switzerlandgregor.sommer@hirslanden.ch (G.S.); 2 Prostate Cancer Center, Hirslanden Klinik St. Anna, 6006 Lucerne, Switzerland; 3 Urology St. Anna, 6006 Lucerne, Switzerland; 4 Center for Oncology, Klinik St. Anna, 6006 Lucerne, Switzerland

**Keywords:** ^18^F-PSMA-1007, furosemide, PET/CT

## Abstract

**Background/Objectives**: ^18^F-PSMA-1007 is one of the more widely used radioligands in prostate cancer imaging with PET/CT. Its major advantage lies in the low urinary tracer activity due to primarily hepatobiliary clearance, but unexpectedly high tracer accumulation in the bladder can occur, potentially hindering assessment of lesions near the prostate bed. This study assesses the impact of furosemide on ^18^F-PSMA-1007 tracer accumulation in the bladder. **Methods**: In this single-center, retrospective, intra-individual comparative analysis, 18 patients undergoing two consecutive ^18^F-PSMA-1007 PET/CT scans for biochemical relapse (BCR) or persistence (BCP)—one with and one without prior furosemide administration—were included. Images were acquired 60 min post-injection of 250 MBq of tracer activity. Standardized Uptake Values (SUVmax, SUVpeak, SUVmean) were measured in the bladder and in tissues with physiological uptake by three readers. Differences were analyzed using Wilcoxon signed-rank tests. The inter-reader agreement was assessed using intraclass correlation coefficient. **Results**: Furosemide significantly decreased bladder SUVmax, SUVpeak, and SUVmean (all *p* < 0.001). Mean bladder SUVmax decreased from 13.20 ± 10.40 to 3.92 ± 3.47, SUVpeak from 10.94 ± 8.02 to 3.47 ± 3.13, and SUVmean from 8.74 ± 6.66 to 2.81 ± 2.56, representing a large effect size (r ≈ 0.55). Physiological tracer uptake in most organs was not significantly influenced by furosemide (all *p* > 0.05). **Conclusions**: Despite the predominantly hepatobiliary clearance of ^18^F-PSMA-1007, furosemide-induced forced diuresis leads to a significant reduction in tracer activity in the bladder, which in clinical practice could help in early detection of tumor recurrence.

## 1. Introduction

Diagnostic imaging has a key role in staging and follow-up assessment of patients diagnosed with prostate cancer. Positron Emission Tomography/Computed Tomography (PET/CT), combining functional and anatomical information with the use of radionuclides targeting the prostate-specific membrane antigen (PSMA), is established for primary staging in prostate tumors with high-risk features, and for biochemical relapse (BCR) or persistence (BCP) [1,2]. PSMA-PET/CT surpasses traditional staging methods with CT and bone scintigraphy in sensitivity, specificity, and the ability to detect lymphatic regional or distant metastases as well as organ metastases, with less equivocal findings and less radiation exposure [3,4]. Several PSMA-targeting radioligands are currently available and used. Among them, ^18^F-PSMA-1007 stands out for being the only radioligand with mostly hepatobiliary clearance [5]. Its low urinary excretion and decreasing bladder activity over time potentially help to delineate prostate cancer lesions located near the bladder [6,7], thereby improving the assessment of local tumor extent. Still, there is urinary excretion, and some patients present unexpectedly high tracer accumulation in the bladder, which is not well understood and seemingly unrelated to imaging or clinical characteristics [8,9]. A general recommendation on when to use hydration and forced diuresis to reduce tracer accumulation in the urinary bladder in PSMA-PET/CT does not yet exist [10]. Our institution has recently changed its protocol for logistic reasons, which resulted in discontinuing the use of furosemide in PSMA-PET/CT with ^18^F-PSMA-1007. However, since the change, we have noticed increased urinary radioactivity and difficult-to-interpret findings due to local tracer accumulation in or close to the prostate bed in the urinary bladder. Our retrospective study aims to assess and quantify the impact of furosemide on ^18^F-PSMA-1007 tracer accumulation in the bladder, its influence on other physiological and non-physiological uptake, and the presence of associated equivocal findings. Our findings shall provide additional guidance for protocol setup in institutions that use ^18^F-PSMA-1007 as a PET tracer and may help enhance the image quality and diagnostic certainty in prostate cancer imaging and theranostics.

## 2. Materials and Methods

This study is a single-center, retrospective, intra-individual comparative analysis designed to assess the impact of furosemide on the urinary activity of ^18^F-PSMA-1007 in PET/CT. It was conducted at Hirslanden Klinik St. Anna in Lucerne, a tertiary care center for prostate cancer in central Switzerland. All procedures adhered to the Swiss Human Research Act and were approved by the regional ethics committee [Ethikkommission Nordwest- und Zentralschweiz (EKNZ), 2024-01504].

### 2.1. Patient Population

The institution’s local database was screened for patients with prostate cancer who underwent two consecutive PET/CT scans using ^18^F-PSMA-1007 for BCR or BCP between January 2020 and the introduction of a new PET/CT scanner in November 2023. Only patients who signed our institution’s research consent form were included. Patients with prior diuretic use or urinary tract surgery affecting bladder anatomy (other than transurethral prostate resection (TUR-P) or radical prostatectomy) were excluded. The target number of patients to be included was ≥20, based on a sample size calculation with a statistical power of 80% and an alpha error of 5%. The expected effect size and variance were derived from a similar trial with ^68^Ga-PSMA-11 and ^18^F-DCFPyL [11].

### 2.2. Radiopharmaceutical

The radiopharmaceutical assessed in this study was ^18^F-PSMA-1007. The ligand was produced at the Center for Radiopharmacy of the University Hospital in Zurich. Radiolabeling with 18-Fluoride also occurred at University Hospital Zurich, Switzerland. The final product was delivered to our clinic on time by special transport and passed quality controls.

### 2.3. Imaging Protocol

PET/CT imaging was performed using a commercially available scanner (Biograph mCT 128, Siemens Healthineers, Knoxville, TN, USA). The image acquisition and furosemide administration were carried out following the local protocol in a standardized manner, following current guidelines [10,12]. The only protocol change between the two groups was the inclusion or exclusion of furosemide administration. Patients were given a protocol-defined fixed activity of 250 MBq bolus injection of ^18^F-PSMA-1007 and scanned after a standardized uptake time of 60 min. In scans with prior furosemide administration, patients received a fixed dose of 20 mg of furosemide intravenously immediately before tracer administration, followed by 500 mL of sodium chloride (NaCl 0.9%) for hydration. The PET scan covered the entire body from the top of the skull to mid-thigh in 3-dimensional mode, with an emission time of 2 min per bed position. All examinations included a low-dose CT scan for attenuation correction and anatomic reference. A diagnostic contrast-enhanced CT scan of the chest and abdomen in the portal venous phase was also part of the standard protocol but was omitted in cases where a corresponding short-term preliminary scan was available.

### 2.4. Data Analysis

Images were analyzed using the dedicated evaluation software provided by the system manufacturer (SyngoVia, Siemens Healthineers, Knoxville, TN, USA). The maximum, mean and peak Standardized Uptake Values (SUVmean, SUVmax, and SUVpeak) were determined by defining volumes of interest (VOIs) in the bladder, blood pool, salivary glands, liver, kidneys, spleen, small bowel, muscle, and bones. An MD candidate performed the primary assessment, and all measurements were independently controlled by two double board-certified specialists in nuclear medicine and radiology with 5 (AK) and 10 (GS) years of experience. For statistical analysis, the mean of the individual measurements of the three readers was used to ensure the accuracy of measurements and reduce individual interpretation bias. Additionally, all corresponding imaging reports were reviewed to identify differences in the number of equivocal findings between the two sets of imaging conditions.

### 2.5. Statistical Analysis

The differences in SUVmax, SUVpeak, and SUVmean in the bladder between the intervention group with prior furosemide administration and the control group without prior furosemide administration were analyzed. Due to the limited sample size and non-normally distributed data, statistical analysis was performed using non-parametric tests to obtain more robust results and reduce the influence of outliers. As a secondary analysis, the differences in SUVmax, SUVpeak, and SUVmean for physiologic tracer uptake were analyzed using Wilcoxon signed-rank tests as well.

The quality of the measurements was assessed using intraclass correlation coefficient (ICC) for the differences in SUV measurements between readers.

The frequency of equivocal findings observed with and without administration of furosemide is reported in absolute numbers, as the study population chosen for the primary research question is undersized for a statistical analysis of this effect.

All statistical analyses were conducted using SPSS software (version 30.0, IBM Corporation, Armonk, NY, USA), with a significance level set at *p* < 0.05. The results were reported with Standard Deviation or 95% confidence intervals to ensure the robustness of the findings.

## 3. Results

### 3.1. Characteristics of the Study Population

A total of 42 patients were identified who underwent an ^18^F-PSMA-1007 PET/CT scan for BCR or BCP without furosemide administration after the protocol change and before the introduction of the new-generation PET/CT scanner. Among these, 20 patients had a prior scan with furosemide, performed on the same PET/CT system using an otherwise identical protocol. Two patients were excluded due to refusal of informed consent, leaving eighteen eligible patients. All patients included had no history of urinary tract surgery or diuretic medication use. Patient characteristics are summarized in Table 1. While the overall dataset was largely complete, kidney function data was unavailable for one patient, and PSA values were missing for one scan with and three scans without furosemide. These specific missing values were excluded from the descriptive statistics.

There were no relevant differences between the intervention (with furosemide) and control (without furosemide) scans regarding the prostate-specific antigen (PSA) levels, the prevalence of local tumor manifestation close to the bladder, renal function, or administered activity, thereby confirming the comparability of both scan conditions in this intra-individual design (Table 1).

The inter-reader agreement for SUV measurements overall and in the bladder was high, with intraclass correlation coefficients for single measures of 0.972 (95% CI [0.969–0.975]) and 0.997 (95% CI [0.996–0.998]) and for average measures of 0.990 (95% CI [0.989–0.991]) and 0.999 (95% CI [0.999–0.999]), respectively (Table 2).

### 3.2. Urinary Bladder Activity

**Figure 1:**
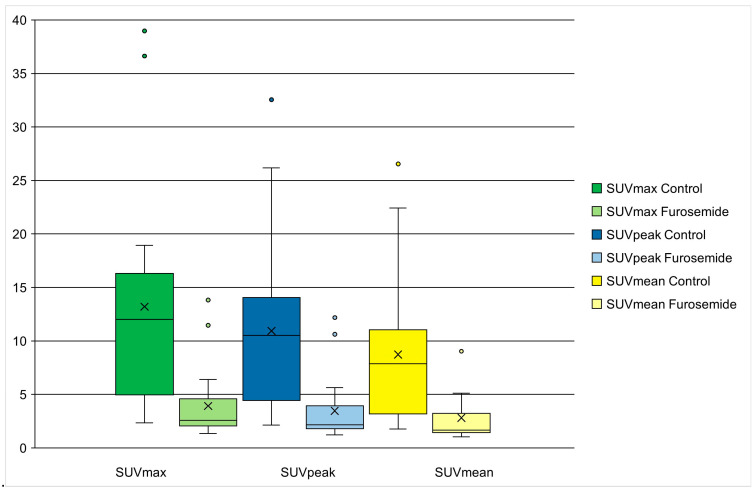
Bladder SUVmax and SUVmean between scans with and without furosemide.

Average SUVmax, SUVpeak, and SUVmean were 13.20 ± 10.40, 10.94 ± 8.02, and 8.74 ± 6.66 for the control image and 3.92 ± 3.47, 3.47 ± 3.13, and 2.81 ± 2.56 for the scan with prior furosemide administration, as illustrated for SUVmax and SUVmean in Figure 1. Due to outliers, the Shapiro–Wilk tests indicated a significant deviation from normality (*p* < 0.05) for the differences in bladder SUV. The Wilcoxon signed-rank test calculated a mean intra-individual decrease in SUV of 9.28 (95% CI [4.24–14.33]), 7.47 (95% CI [3.47–11.46]), and 5.93 (95% CI [2.64–9.23]) in scans taken 60 min after the tracer and furosemide injection, representing a significant difference in tracer activity in the bladder (*p* < 0.001), with a large effect size (r of 0.55 for SUVmax, peak and mean, respectively), as shown in Table 3.

### 3.3. Physiological Uptake in Other Tissues

**Figure 2:**
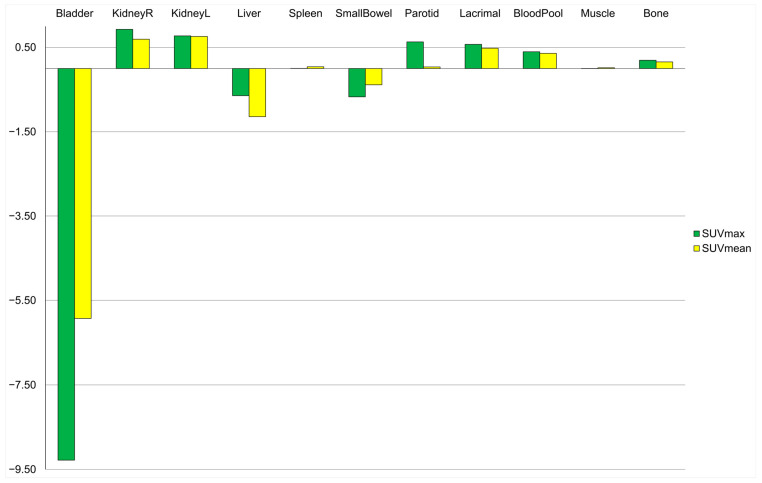
Difference in SUVmax and SUVmean between control and furosemide.

Physiological tracer uptake in most organs was not significantly influenced by furosemide (all *p* > 0.05), although small but statistically significant increases in SUVmax after furosemide administration were observed in the blood pool and bone (increase in SUVmax of 0.40 (95% CI [0.02–0.77]) and 0.19 (95% CI [0.05–0.34]), respectively; all *p* < 0.05), as shown in Figure 2 and Table 3.

### 3.4. Equivocal Findings

**Figure 3:**
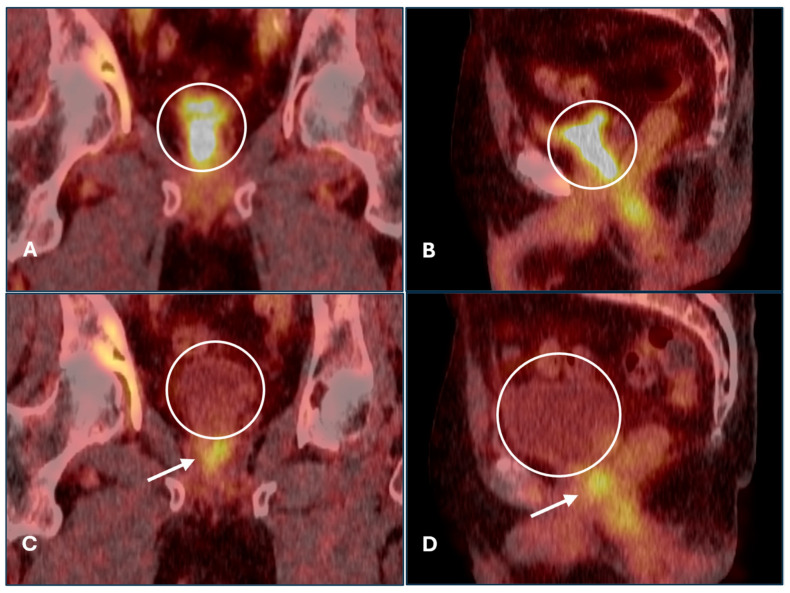
Illustrative case description of potential effect of furosemide for assessment of local tumor recurrence. ^18^F-PSMA-1007 PET/CT in a patient with disseminated prostate cancer (lymphogenic and osseous metastases), status post 6 cycles of palliative chemotherapy. Without furosemide (**A**,**B**), high tracer accumulation in the bladder (circle) obscures possible residual tumor at the bladder neck seen in the earlier scan with furosemide (arrows in (**C**,**D**)). Accurate detection of this finding may be of low relevance in metastatic disease but critical in early BCR.

Tumor tissue near the bladder was seen in 7/18 (39%) cases in the furosemide group and 6/18 (33%) cases in the control group. However, the number of findings that were considered equivocal according to the reports was limited to two cases, one of which is shown in Figure 3, illustrating the potential benefit furosemide can offer.

## 4. Discussion

This intra-individual comparative study aimed to evaluate the impact of furosemide on ^18^F-PSMA-1007 activity in the bladder, its influence on physiological tracer uptake in other tissues, and its potential effect on equivocal findings. Our results demonstrate a significant reduction in SUVmax, SUVpeak, and SUVmean values compared to the control group. The observed 68–70% decrease in SUV is surprisingly large, given that the renal clearance of ^18^F-PSMA-1007 is only about 8% [5]. Previous studies by Dang et al. [8] and Allach et al. [9] found that up to 25% of patients undergoing ^18^F-PSMA-1007 PET scans exhibit unexpectedly high urinary tracer activity, indicating that this phenomenon is common but not yet well understood. The effect of furosemide on tracer activity in the bladder has only been studied for tracers with renal clearance. Here, furosemide leads to a strong reduction in intravesical radioactivity (Cohen’s d of 0.8–0.9 [11]) for the tracers ^68^Ga-PSMA-11, ^68^Ga-PSMA-I&T and ^18^F-DCFPyL compared to no premedication or hydration only [11,13,14], without affecting physiologic tracer uptake in other tissues [11,15]. This was found to improve image quality, tumor delineation, and reporter confidence [15,16]. Thereby, it might also increase the detection rate at low PSA values [15,17]. A large reduction in urinary tracer activity after furosemide administration with improved visualization of pelvic structures was also observed for ^18^F-Fluorodeoxyglucose (FDG)-PET/CT, although the inhomogeneity of the published studies (hydration protocol variations and only one direct comparison of hydration alone versus hydration with diuretics) makes interpretation difficult [18,19,20,21].

Physiological tracer uptake in most organs analyzed in our study was not significantly influenced by furosemide administration, and overall tracer biodistribution was similar to previous data on ^18^F-PSMA-1007 [8,22]. This aligns with comparable results reported for ^68^Ga-PSMA-11, showing no impact of forced diuresis on physiological tracer uptake in other tissues [15]. However, small but statistically significant increases in mean tracer activity were observed in the blood pool and bone. As no convincing potential confounders were identified, the reason for this unexpected minor difference remains unclear, but it is unlikely to affect image interpretation significantly.

The current guidelines for PSMA-PET/CT are somewhat heterogeneous regarding the use of furosemide or specific hydration protocols. The 2023 Procedure Guideline for Prostate Cancer Imaging with PSMA-ligand PET/CT by Afshar-Oromieh et al. [10] recommends bladder emptying immediately before image acquisition and suggests that hydration or diuretic use might help for PSMA tracers with renal clearance, while the European Association of Nuclear Medicine (EANM)/Society of Nuclear Medicine and Molecular Imaging (SNMMI) guideline [12] similarly highlights the benefit of furosemide with renally cleared PSMA tracers to decrease the number of false-negative findings for lesions adjacent to the bladder. In contrast, the EANM Focus 5 [23] does not address diuretic use at all. Guideline recommendations regarding the use of diuretics in imaging protocols for ^18^F-PSMA-1007 currently do not exist. For FDG-PET/CT, the EANM Procedure Guideline judges pre-hydration alone to be sufficient to avoid most potential reading errors but says that furosemide administration could be considered for small pelvic tumors [24].

Our findings suggest that forced diuresis can significantly reduce urinary tracer levels with ^18^F-PSMA-1007, potentially improving lesion detection near the bladder and minimizing equivocal findings, as illustrated in our case description in Figure 3. As perianastomotic and retrovesical regions are common sites for recurrence post prostatectomy [25,26], differentiating pathological uptake from urinary activity in this area is crucial. Particularly in patients with early BCR, equivocal findings may lead to inappropriate management or delayed therapy. The number of equivocal findings in our study was low, due to the small sample size. Tumor tissue near the bladder was seen in approximately one third of our cohort, which is slightly higher than the local recurrence rates of 20–30% reported in other studies [11,14,15,20,21,25,26].

As a retrospective analysis, our study is limited by the availability and quality of existing data. Patient selection was constrained by a protocol change and the installation of a new PET/CT scanner, resulting in a narrow inclusion window of only 6 months. The small sample size limits statistical power particularly for the secondary endpoints like the frequency of equivocal findings and, therefore, we cannot derive definitive conclusions regarding the impact of furosemide on reader confidence and clinical decisions in ^18^F-PSMA-1007 PET/CT from our data. A major strength of our study, however, lies in its intra-individual design. By using each patient as his own control, undergoing two PET/CT scans under virtually identical conditions except for furosemide administration, we minimized bias due to individual differences in radiopharmaceutical biodistribution and excretion. Review by two double-board certified specialists also reduced interpretation bias. Thus, despite the small number of cases, sufficient statistical power was ultimately achieved for the main research question of the study.

**Figure A1:**
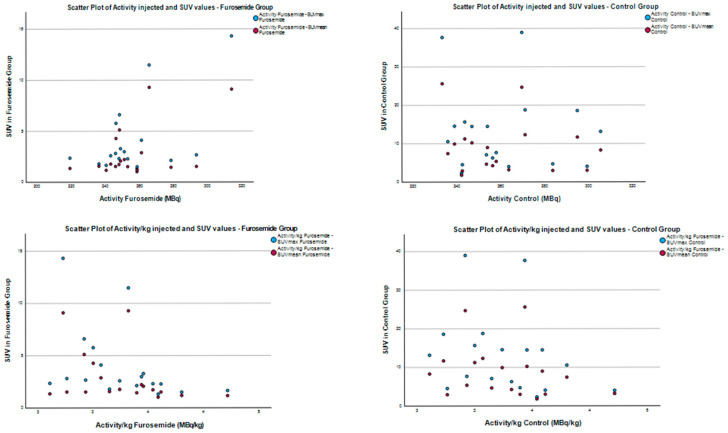
Descriptive Analysis with no correlation between Activity and Bladder SUV.

A potential confounding factor from a technical point of view is the dosage of the furosemide that has been administered in a protocol-defined fixed standard dose of 20 mg, as its effect on the kidneys is dose-dependent and influenced by renal function [27,28]. The fixed furosemide dose and the small sample size with overall good kidney function with low inter-individual variance in eGFR made underdosing unlikely and precluded any meaningful dose–effect analysis. Another technical issue is variations in injected tracer activity (absolute and per kg) that also existed in our cohort due to technical reasons during the manual administration process, but were not significantly different between scans, and did not correlate with bladder activity (Figure A1 in the Appendix). Finally, we used a single time-point PET acquisition with a standardized uptake time of 60 min, which limits our understanding of the impact of furosemide on bladder activity and image quality at different time-points. Guidelines do not recommend an optimal acquisition time for ^18^F-PSMA-1007 [10,12]. While imaging 60 min post-injection is performed frequently for practical reasons, there is some evidence in the literature that delayed imaging ≥90 min post-injection could improve image quality and decrease bladder activity [7,29,30]. However, the clinical relevance of this remains controversial: while earlier studies comparing 60 and 180 min [22] and a mean of 94 versus 144 min [31] did not find a difference in the number of lesions, Hvittfeldt et al. focused in their study explicitly on the optimal uptake time for tumor detection in a larger patient cohort and found more pathologic lesions at 120 min compared to 60 min post-injection [30]. Relt et al., on the other hand, could not even find a correlation between urinary SUV and time after injection [32]. In any case, high urinary tracer activity with ^18^F-PSMA-1007 is a regularly observed phenomenon also at later imaging times and seemingly unrelated to standard scan parameters [8,9]. Therefore, furosemide may still be helpful to reduce activity in the bladder in more delayed imaging. As other studies using delayed PET imaging raise concerns that the effect of furosemide might diminish over time, as found with ^68^Ga-PSMA-11 and ^18^F-DCFPyL [13,33], delayed furosemide administration may be an option. Ultimately, the ideal timing for image acquisition and furosemide injection in PSMA-PET/CT remains an open question.

## 5. Conclusions

Despite the low renal clearance of ^18^F-PSMA-1007, furosemide-induced forced diuresis leads to a significant decrease in bladder tracer activity in ^18^F-PSMA-1007 PET/CT that might be relevant for early detection of tumor recurrence in the perianastomotic and retrovesical regions. Larger prospective studies are required to determine and quantify its potential benefit for diagnostic accuracy and its impact on clinical management and treatment decisions.

## Figures and Tables

**Table 1:** Patient characteristics (N = 18).

Patient Characteristics	Occurrence	Mean	STD	Min	Max
Gleason Score					
7	7				
8	4				
9	7				
PSA Furosemide		11.0	20.5	0.2	78.0
PSA Control		32.7	78.2	0.0	300.0
Age at First Scan		72.3	6.7	59.3	81.7
eGFR Furosemide ^1^		81.8	11.4	52.2	90.0
eGFR Control ^1^		75.8	12.1	60.2	90.0
Injected Activity Furosemide		255.9	21.6	219.0	313.8
Injected Activity Control		260.2	22.4	232.9	305.1
Injected Activity/kgBW Furosemide		3.3	0.4	2.6	4.2
Injected Activity/kgBW Control		3.4	0.5	2.6	4.6
Time between Scans		15.7	10.9	3.0	43.0

STD (Standard Deviation), Min (lowest value), Max (highest value), PSA (prostate-specific antigen) [ng/mL], age [years], eGFR [mL/min/1.73 m^2^], activity [MBq], Activity/kgBW (kilograms of bodyweight) [MBq/kg], time between scans [months]. ^1^ estimated Glomerular Filratation Rate (eGFR) > 90 for statistical calculations treated as 90.

**Table 2:** Inter-reader agreement analysis using intraclass correlation coefficient.

	Intraclass Correlation ^b^	95% CI	Significance
Bladder Single Measures	0.997 ^a^	0.996–0.998	<0.001
Bladder Average Measures	0.999 ^c^	0.999–0.999	<0.001
Overall Single Measures	0.972 ^a^	0.969–0.975	<0.001
Overall Average Measures	0.990 ^c^	0.989–0.991	<0.001

Two-way mixed-effect model where people effects are random and measure effects are fixed. a: The estimator is the same, whether the interaction effect is present or not. b: Type A intraclass correlation coefficients using an absolute agreement definition. c: This estimate is computed assuming the interaction effect is absent, because it is not estimable otherwise.

**Table 3:** Wilcoxon signed-rank test for the differences in SUV values between control and furosemide scans.

	SUVmax		SUVpeak		SUVmean	
Differences in SUV	Significance ^a^ (p)	Effect Size (r)	Significance ^a^ (p)	Effect Size (r)	Significance ^a^ (p)	Effect Size (r)
Bladder	0.0003	0.548	0.0003	0.550	0.0003	0.548
Kidney Right	0.468	0.127	0.393	0.150	0.279	0.185
Kidney Left	0.609	0.091	0.766	0.050	0.551	0.105
Liver	0.523	0.113	0.609	0.090	0.067	0.309
Spleen	0.823	0.040	0.823	0.040	1.000	0.004
Small Bowel	0.369	0.156	0.393	0.150	0.442	0.134
Parotid Gland	0.865	0.033	0.766	0.050	0.899	0.025
Lacrimal Gland	0.304	0.178	0.246	0.200	0.196	0.221
Blood Pool	0.016	0.396	0.021	0.380	0.008	0.432
Muscle	0.790	0.047	0.890	0.030	0.279	0.185
Bone	0.024	0.370	0.012	0.410	0.007	0.436

a: Exact significance (two-tailed).

## Data Availability

The raw data supporting the conclusions of this article will be made available by the authors on request.

## Abbreviations

The following abbreviations are used in this manuscript:

PET/CT	Positron Emission Tomography/Computed Tomography
PSMA	Prostate-specific membrane antigen
BCR	Biochemical Relapse
BCP	Biochemical Persistence
18F	18-Fluor-
TUR-P	Transurethral Prostate Resection
SUVmax	Maximum Standardized Uptake Value
SUVpeak	Peak Standardized Uptake Value
SUVmean	Mean Standardized Uptake Value
VOI	Volume of Interest
ICC	Intraclass Correlation Coefficient
MD	Medical doctorate
kgBW	Kilogram of bodyweight
PSA	Prostate specific antigen
eGFR	estimated Glomerular Filtration Rate
FDG	Fluorodeoxyglucose
EANM	European Association of Nuclear Medicine
SNMMI	Society of Nuclear Medicine and Molecular Imaging
